# Biomechanical evaluation of In-Out-In pedicle screws for atlantoaxial posterior fixation in the presence of high-riding vertebral artery and narrow C2 pedicles: a finite element analysis

**DOI:** 10.1186/s13018-025-06095-2

**Published:** 2025-07-16

**Authors:** Xiong-han Lian, Wen He, Wen-jia Sun, Xu-chao Lin, Huo-huo Xue, Zhi-feng Zeng, Yu-fan Chen, Ming-di Chen, Feng-guang Wei, Zhi-qiang Wang, Hong-liang Ruan, Zi-qi Luo, Xiang-ting Chen, Xin Chen, Jing-lai Xue

**Affiliations:** 1 Fuzhou Second General Hospital, Fuzhou, 350007 China; 2 Fujian University of Traditional Chinese Medicine, Fuzhou, 350007 China

**Keywords:** Atlantoaxial, Posterior fixation, Pedicle screw, Biomechanics, Finite element analysis

## Abstract

**Introduction:**

In patients with high-riding vertebral artery (HRVA) and narrow pedicles, placement of C2 pedicle screws carries a significant risk of vertebral artery injury. The in-out-in pedicle screws (IOIPS) technique offers an alternative posterior fixation strategy. However, the biomechanical performance remains unclear.

**Methods:**

A finite element model of the upper cervical spine (C0-C2) was developed to simulate HRVA and narrow pedicles. All posterior fixation constructs utilized C1 pedicle screws, while four different techniques were applied to C2: IOIPS, pedicle screws, pars screws, and translaminar screws. A vertical load of 50 N and a torque of 1.5 Nm were applied at C0 to assess and compare the range of motion (ROM) and stress distribution.

**Results:**

All four fixation techniques effectively reduced ROMs at the atlantoaxial, with IOIPS demonstrating the lowest ROMs and translaminar screws showing the highest. Pedicle and pars screws exhibited higher von Mises stresses within the implants, while IOIPS achieved a more uniform stress distribution. Translaminar screws had the lowest implant stresses but provided the least stability.

**Conclusion:**

For patients with HRVA and narrow C2 pedicles, IOIPS offers superior stability along with relatively low and evenly distributed implant stress, suggesting it is a biomechanically viable fixation technique. However, its application should be reserved for carefully selected cases rather than adopted as a routine surgical strategy.

## Introduction

Atlantoaxial instability, involving the articulation between the atlas (C1) and axis (C2), is a critical condition resulting from trauma, congenital anomalies, inflammatory diseases, or neoplastic processes. Stabilization of the C1-C2 complex is essential for preventing neurological injury, providing structural support, and achieving long-term fusion. Posterior fixation has gained popularity due to the robust biomechanical stability and favorable clinical outcomes.

However, the presence of high-riding vertebral artery (HRVA) and narrow C2 pedicles presents a significant challenge in safely and effectively placing C2 pedicle screws, increasing the risk of vertebral artery injury during insertion [1, 2]. HRVA and narrow C2 pedicles are commonly encountered in congenital or developmental abnormalities, with a prevalence of approximately 24.52% in Europeans [3]. Traditional C2 pedicle screw placement techniques may be unsuitable or risky for patients with these anatomical variations. Common alternatives include pars screws, translaminar screws, and others [4].

Du innovatively applied the in-out-in pedicle screw (IOIPS) technique to the cervical spine, effectively overcoming anatomical constraints [5]. This technique involves a unique C2 trajectory, where the pedicle screw is shifted medially, exiting through the medial cortical bone of the isthmus, then proceeding along the outer edge of the spinal canal before re-entering the vertebral body. This pathway bypasses high-risk areas, improving safety and stability. However, the biomechanical performance is still unclear.

Finite element (FE) analysis has become an invaluable tool for assessing spinal biomechanics and implant performance under various loading conditions. This study aims to evaluate the biomechanical performance of the IOIPS technique for atlantoaxial posterior fixation in the presence of HRVA and narrow C2 pedicles. By comparing the range of motion (ROM) and stress distribution among the IOIPS and conventional techniques, the study seeks to guide surgical decision-making and improve clinical outcomes.

## Methods

### Construction of FE model

**Fig. 1:**
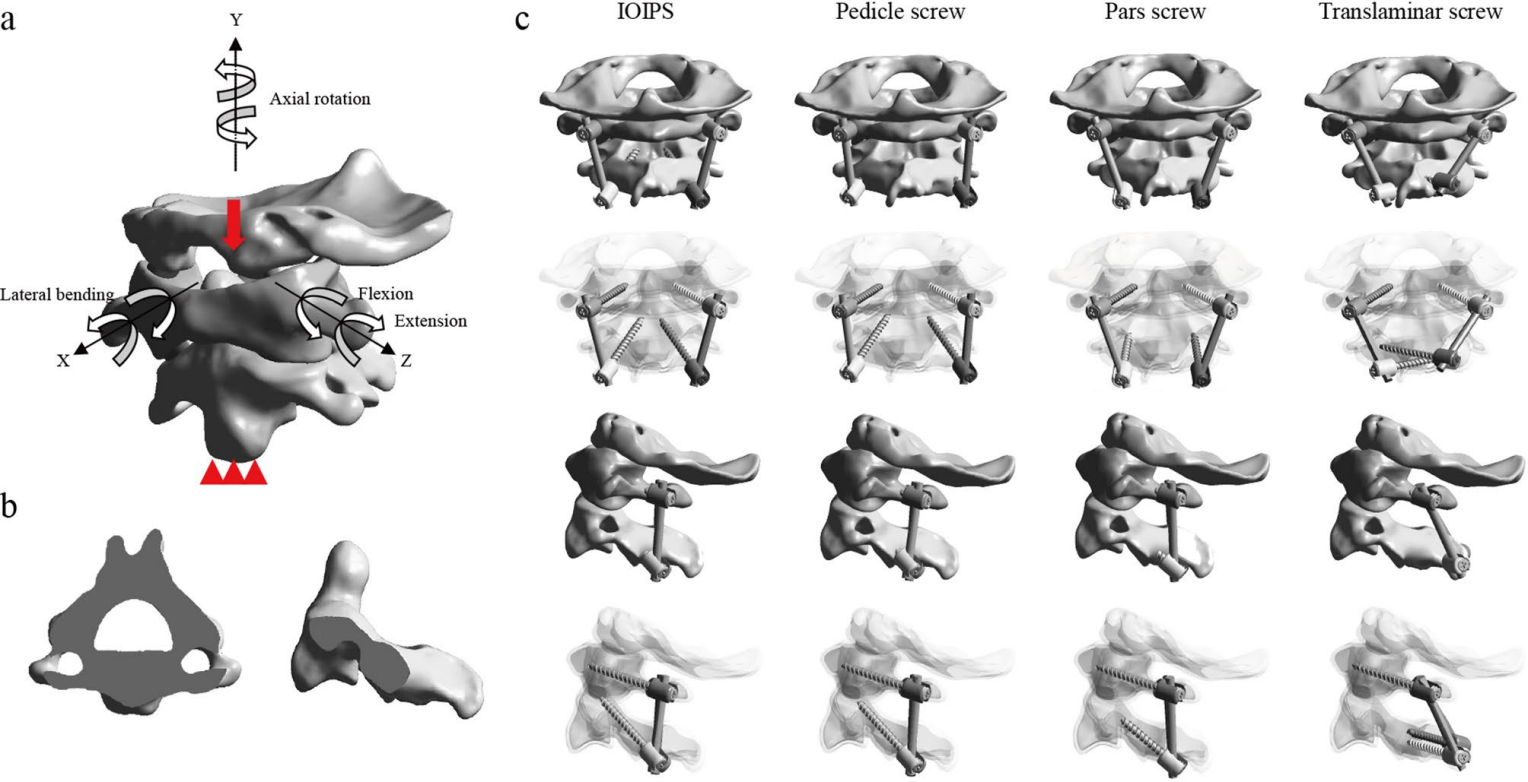
**a** The intact FE model with boundary and loading conditions; **b** Section of C2 with high-riding vertebral artery and narrow pedicles; **c** Different posterior atlantoaxial fixation surgeries

The high-resolution, continuous thin-slice 3D CT imaging data were obtained from a healthy male volunteer (age: 52 years; height: 168 cm; weight: 70 kg; supine position; no cervical-related diseases). Ethical approval was granted by the Institutional Review Board of Fuzhou Second General Hospital, and informed consent was obtained from the participant. First, the C0-C2 bony structures were extracted from the CT scans using Mimics (version 25.0; Materialise, Leuven, Belgium). The surfaces were then smoothed and segmented in Geomagic (version 2024; 3D Systems, Research Triangle Park, NC, USA). Next, the cortical bone, cancellous bone, articular cartilage, and implant system were modeled and assembled in SOLIDWORKS (version 2024; Dassault Systems, Waltham, MA, USA). The C1 and C2 vertebrae consist of an outer layer of cortical bone and an inner core of cancellous bone, with a cortical bone thickness of 1.5 mm. The C0 is entirely composed of cortical bone [6, 7]. The HRVA was diagnosed based on a C2 isthmus height (C2IsH) ≤ 5 mm and/or a C2 internal height (C2InH) ≤ 2 mm, measured 3 mm lateral to the lateral border of the spinal canal on sagittal CT images. We simulated type I HRVA (C2IsH ≤ 5 mm and normal C2InH) because it is the most common clinical condition (78.2%) [8]. To construct the FE model with type I HRVA, the transverse foramen was slightly adjusted to ensure that the pedicle could accommodate only a single screw (Fig. 1b). Finally, the ligamentous complexes were simulated in ANSYS (version 2024 R2; ANSYS Inc., Canonsburg, PA, USA). All bones were connected by ligaments at the corresponding anatomical attachment sites, including the anterior longitudinal ligament, anterior atlantooccipital membrane, tectorial membrane, posterior atlantooccipital membrane, posterior atlantoaxial membrane, joint capsules, alar ligament, apical ligament, transverse ligament, and cruciate ligament-vertical portion [6, 7]. Additionally, unstable models were simulated by removing the transverse ligament [6, 7]. The material properties used in the models are summarized in Table 1 [6, 7, 9, 10].

**Table 1:** Spinal structure and instrumentation material properties

Components	Element type	Young’s modulus (MPa)	Poisson’s ratio
Titanium Cortical bone	C3D4 C3D4	110,000 12,000	0.30 0.29
Cancellous bone Cartilage Alar ligament Transverse ligament Other ligaments and joint capsules	C3D4 C3D8 T3D2 4-node membrane elements T3D2	450 10 5 20 10	0.29 0.30 0.30 0.30 0.30

### Posterior implant

The implants used in the FE models included screws, rods, and nuts. The diameters of both the screws and rods were 3.5 mm [7, 9–11]. Pedicle screws were implanted in the C1 in all models. Four different screw placement techniques were applied to the C2: IOIPS, pedicle screws, pars screws, and translaminar screws (Fig. 1c) [4, 5, 7]. The IOIPS technique is achieved by medializing the entry point of the pedicle screw and increasing the lateral angulation. The screw lengths were 30 mm for IOIPS, pedicle screws, and translaminar screws, and 20 mm for pars screws.

### Boundary, loading, and connection conditions

To isolate the effect of surgical technique, consistent boundary and loading conditions were applied across all models. The inferior surface of the C2 vertebral body was fully constrained in all directions. A vertical load of 50 N and a torque of 1.5 Nm were applied at C0 to simulate physiological head motion (Fig. 1a) [7, 10, 11]. The facet joint interactions were defined as frictional contacts with a coefficient of friction of 0.1 [6, 12, 13]. Frictional contact was also modeled between the bone and screws, with a friction coefficient of 0.3 [10, 14], to simulate realistic bone-screw integration.

### Convergence analysis

While increasing mesh density enhances accuracy, it also increases computational cost. After evaluating four mesh sizes (2.0 mm, 1.5 mm, 1.0 mm, and 0.5 mm), a mesh size of 1.0 mm was selected, as it offered an optimal balance between accuracy and efficiency. The corresponding peak variation in von Mises stress was less than 5% [9], ensuring reliability with reasonable computational time (Table 2).

**Table 2:** Convergence analysis results

Mesh size	Unit	Node	Peak von Mises stress variation
2.0 mm	72,160	132,193	>5%
1.5 mm	97,019	175,374	>5%
1.0 mm	166,586	293,972	<5%
0.5 mm	393,753	697,167	<5%

## Results

### Validation

The validity of the model was confirmed by calculating the ROMs of the C1-C2 segment and comparing them with previously published FE analyses and in vitro experiments [7, 15–18]. Both the intact and unstable FE models developed fell within the standard error range (Table 3), indicating the suitability for further analysis.

**Table 3:** Comparison of the C1-C2 ROMs with previously published data

ROM (°)	Research	Flexion	Extension	Lateral Bending	Axial Rotation
Intact	Panjabi	11.5 ± 2.0	10.9 ± 1.1	4.0 ± 0.8	38.3 ± 1.7
	Cai	11.7	9.5	4.1	38.7
	Zhang	12.5	12.2	5.0	28.5
	Shao	10.46	10.56	5.16	24.50
	Current study	12.06	11.13	4.56	30.80
Unstable	Cai	14.2	·12.7	6.2	45.1
	Zhang	16.9	14.2	5.2	30.1
	Shao	14.00	12.24	7.50	29.30
	Current study	15.88	13.98	6.54	35.12

### The ROMs of the surgical models

**Fig. 2:**
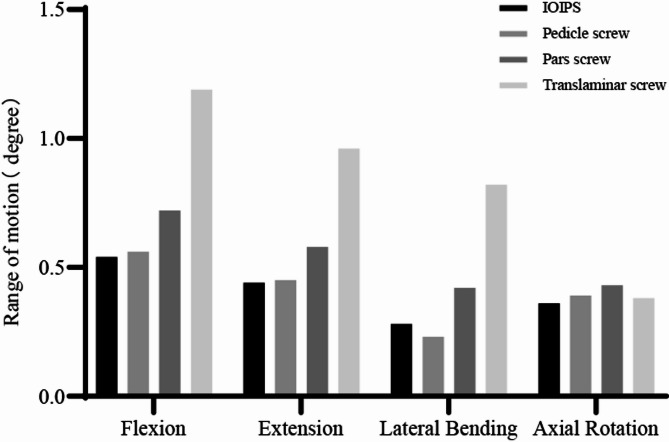
The ROMs of the postoperative models

FE analysis was performed to evaluate the ROMs associated with four posterior fixation techniques: IOIPS, pedicle screws, pars screws, and translaminar screws, under four physiological motion conditions: flexion, extension, lateral bending, and axial rotation (Fig. 2). In flexion, the translaminar screws exhibited the highest ROM (1.19°), indicating the least restraint among the four constructs, followed by the pars screws (0.72°), pedicle screws (0.56°), and IOIPS (0.54°). A similar trend was observed during extension, where the translaminar screws again demonstrated the highest ROM (0.96°), while IOIPS showed the lowest ROM (0.44°). For lateral bending, the translaminar screws resulted in a significantly larger ROM (0.82°) compared to the other three configurations. The pars screws showed a moderate ROM of 0.42°, followed by IOIPS (0.28°), and the pedicle screws (0.23°), which exhibited the greatest motion restriction. In axial rotation, the pars screws exhibited the largest ROM (0.43°), while the pedicle screws showed the smallest (0.39°). The IOIPS and translaminar screws presented intermediate values of 0.36° and 0.38°, respectively. Overall, the translaminar screws consistently showed the greatest ROM in flexion, extension, and lateral bending, suggesting relatively lower mechanical stability. In contrast, the IOIPS configuration provided superior biomechanical rigidity across most motion directions.

### Peak von mises stress of implants

**Fig. 3:**
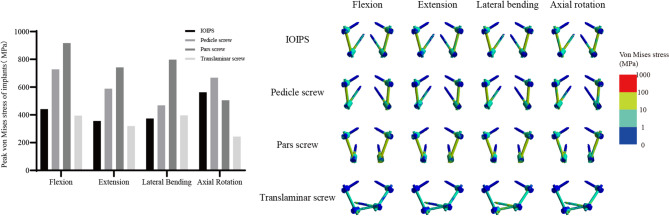
Peak von Mises stresses and stress distribution cloud maps of implants

The peak von Mises stresses experienced by each implant under four motion conditions were evaluated to assess mechanical load distribution and potential risk of implant failure (Fig. 3). In flexion, the highest peak stress was observed in the pars screws (917.40 MPa), followed by the pedicle screws (728.12 MPa), IOIPS (440.86 MPa), and translaminar screws (394.66 MPa). A similar trend was noted in extension, where the pars screws again demonstrated the greatest stress concentration (742.17 MPa), while the translaminar screws exhibited the lowest (319.47 MPa). Under lateral bending, the pars screws remained the most stressed construct (798.15 MPa), and the pedicle screws showed moderate stress (468.60 MPa). The IOIPS (373.44 MPa) and translaminar screws (395.87 MPa) exhibited relatively lower peak stresses. In axial rotation, the pedicle screws demonstrated the highest peak stress (667.75 MPa), followed closely by IOIPS (562.60 MPa) and pars screws (505.83 MPa). The translaminar screws recorded the lowest peak stress (243.57 MPa), indicating the most favorable stress profile under this motion. Overall, the pars screws consistently exhibited the highest peak stresses across most loading conditions, suggesting a higher mechanical demand on this construct and a potentially increased risk of fatigue failure. In contrast, the translaminar screws consistently showed the lowest peak stresses in all directions, indicating better stress distribution and mechanical resilience under physiological loading.

## Discussion

Pedicle screws were commonly utilized in upper cervical spine surgeries due to the superior fixation strength. However, one of the major concerns during screw placement is the risk of vertebral artery injury, given the complex and variable anatomy of the region. The incidence of vertebral artery injury during C2 screw insertion has been reported to be as high as 8.2% [7]. Preoperative computed tomography angiography is considered the gold standard for evaluating the course and variations of the vertebral artery. Notably, while vertebral artery anomalies are relatively common in the general population, their prevalence significantly increases in patients with craniovertebral junction anomalies, such as basilar invagination, atlantoaxial dislocation, and C1 assimilation, even reaching up to 60% [19]. These anatomical variations present substantial challenges for safe screw placement and must be carefully considered during surgical planning.

When C2 pedicle screw placement is not feasible, alternative fixation methods such as C2 pars screws and translaminar screws are commonly employed. However, our FE analysis demonstrated that both pars screws and translaminar screws resulted in significantly increased ROMs under loading conditions compared to standard pedicle screws. These findings are consistent with previous in vitro biomechanical studies and FE research [7, 20–22]. Elliott reported a higher rate of nonunion in patients treated with C2 pars screws (4.4%) compared to those with pedicle screws (0.22%) [23]. Similarly, in the studies by Chang, the success rate of C1-C2 fusion was significantly lower in patients treated with bilateral translaminar screws (57.1%) or unilateral screws (78.9%), compared to bilateral pedicle screws (100%) [24]. These differences may be attributed to the lack of anterior and middle column support provided by these alternative fixation techniques. Therefore, in patients with craniovertebral junction instability accompanied by anatomical variations, particularly those requiring rigid fixation to maintain reduction and achieve solid fusion, a salvage technique that enables long, strong fixation spanning the entire C2 vertebra should be carefully considered.

According to CT-based anatomical studies, the distance from the medial border of the C2 pars to the dura is approximately 3.53 ± 1.57 mm [25], which is sufficient to accommodate a 3.5 mm screw, even in cases of basilar invagination [26]. By carefully understanding the spatial relationship between the bone and the dura, and with the protection of a Penfield dissector, Du first introduced the concept of the C2 IOIPS [5]. This technique involves drilling along the inner cortex of the C2 pars, traversing the lateral edge of the spinal canal, and entering the anterior vertebral body through the trajectory of a C2 modified pedicle screw, thereby achieving tricortical or even quadricortical purchase [5, 27]. In the present study, we evaluated the biomechanical performance of the IOIPS. Due to the long transosseous path through dense bone with multiple anchoring points, as well as the larger lateral insertion angle that enhances resistance to sagittal plane motion, the IOIPS even exhibited stability comparable to or even better than pedicle screws. Under lateral bending, the ROMs of the pedicle screws were slightly lower than those of the IOIPS. This difference may be attributed to the more complete lateral bone support, as well as the advantage of a trajectory that is more perpendicular to the coronal plane, enhancing resistance to lateral bending moments. Even more encouraging is that all existing clinical studies have demonstrated that the IOIPS achieves fusion without causing any vertebral artery injuries or dural tears [5, 27, 28].

In FE analysis, the von Mises stress at the bone-implant interface has significant clinical relevance. It serves as an important indicator for predicting implant stability, optimizing implant design, and preventing postoperative implant-related complications. The bone-implant interface stress increased progressively from IOIPS to pedicle screws and pars screws. Further inspection of the stress contour maps reveals that, in all cases, peak stress is consistently concentrated at the proximal threads, precisely at the screw entry point and the cortical bone interface. Similar stress concentration patterns have been reported in multiple FE studies [7, 29, 30]. The insertion of the screw disrupts the continuity of the native bone architecture, and the stark contrast in material properties between the bone and the implant creates a mechanical discontinuity. As a result, stress transmission undergoes abrupt redirection and becomes highly concentrated around the screw entry site. Fortunately, a portion of the stress is also transmitted along the screw axis and dispersed into the surrounding structures, providing a certain degree of buffering and mitigating excessive stress accumulation at the interface.

Both pedicle screws and pars screws primarily traverse cancellous bone. Under identical loading conditions, the pedicle screw achieves better stress dispersion due to a longer moment arm. Notably, the IOIPS demonstrated the lower peak stress. Although portions of the IOIPS trajectory extend outside the vertebral body, the multiple cortical bone purchase points it establishes serve as robust anchors that mitigate abrupt stress concentrations. The evenly distributed stress patterns of the screw in the maps underscore its superior biomechanical performance. While the translaminar screws exhibited the lowest implant stress among all fixation types, it also corresponded to the greatest ROMs. Low implant stress does not necessarily indicate superior clinical performance; rather, it may reflect inadequate participation of the implant in structural stabilization. Therefore, implant stress must be interpreted alongside ROM and other indicators to comprehensively evaluate biomechanical stability and clinical applicability.

Naturally, our study has several limitations. First, although the model was reconstructed based on actual imaging data, certain simplifications were inevitable. For example, soft tissues such as muscles were not included, and idealized material properties were assumed. These factors may result in discrepancies between simulated and actual biomechanics. Second, the HRVA geometry was generated by repositioning the transverse foramen on a normal C2 model. Although this approach was carefully guided by CT data and existing literature, it was not derived directly from a specific patient, and therefore may not perfectly reflect anatomical reality. Additionally, the ROM values obtained in our FE model were significantly lower than those reported in cadaveric studies [21]. This discrepancy may stem from the idealized simulation of the bone-implant interface in FE analysis, as well as potential structural damage or fatigue that occurred in cadaveric specimens during preparation or repeated biomechanical testing. Lastly, current in vitro studies on IOIPS are still lacking, and high-quality clinical data remain limited. Further biomechanical and clinical research is needed to validate our findings. Therefore, the primary goal of this study is to illustrate biomechanical trends rather than to draw definitive clinical conclusions.

## Conclusion

For patients with HRVA and narrow C2 pedicles, our FE analysis indicates that the IOIPS technique provides excellent biomechanical stability and a more favorable stress distribution, while effectively minimizing the risk of vertebral artery and dural injury. Notably, its biomechanical performance even surpasses that of traditional pedicle screws. However, the clinical efficacy of IOIPS requires further validation through future in vitro experiments and high-quality clinical studies. Given its technical complexity and specific anatomical indications, the use of IOIPS should be approached with caution and should not be adopted as a routine fixation method.

## Data Availability

The datasets used and analyzed during the current study are available from the corresponding author on reasonable request.

## Abbreviations

HRVA High: Riding Vertebral Artery
IOIPS In: Out-In Pedicle Screws
ROM: Range of Motion
FE: Finite Element

## References

[1] Yoshida M, Neo M, Fujibayashi S, et al. Comparison of the anatomical risk for vertebral artery injury associated with the C2-pedicle screw and atlantoaxial transarticular screw. Spine. 2006;31(15):E513–517. 16816753 10.1097/01.brs.0000224516.29747.52

[2] Yeom JS, Buchowski JM, Kim HJ, et al. Risk of vertebral artery injury: comparison between C1-C2 transarticular and C2 pedicle screws. Spine J. 2013;13(7):775–85. 23684237 10.1016/j.spinee.2013.04.005

[3] Klepinowski T, Zylka N, Pala B, et al. Prevalence of high-riding vertebral arteries and narrow C2 pedicles among Central-European population: a computed tomography-based study. Neurosurg Rev. 2021;44(6):3277–82. 33559797 10.1007/s10143-021-01493-6 PMC8592946

[4] Singh DK, Shankar D, Singh N, et al. C2 screw fixation techniques in atlantoaxial instability: A technical review. J Craniovertebr Junction Spine. 2022;13(4):368–77. 36777907 10.4103/jcvjs.jcvjs_128_22 PMC9910137

[5] Du YQ, Yin YH, Qiao GY, et al. C2 medial pedicle screw: a novel in-out-in technique as an alternative option for posterior C2 fixation in cases with a narrow C2 isthmus. J Neurosurg Spine. 2020;33(3):281–7. 32357339 10.3171/2020.2.SPINE191517

[6] Zhang B-c, Liu H-b, Cai X-h et al. Biomechanical comparison of a novel transoral atlantoaxial anchored cage with established fixation technique - a finite element analysis. BMC Musculoskelet Disord 2015; 16(1). 10.1186/s12891-015-0662-7 PMC4579577 26395763

[7] Shao M, Dai Y, Zhu W, et al. Bicortical short C2 Pars screw fixation for High-Riding vertebral artery provided sufficient Biomechanical stability: A finite element study. Spine. 2022;47(4):369–75. 34107525 10.1097/BRS.0000000000004141

[8] Klepinowski T, Żyłka N, Pettersson SD, et al. Types of high-riding vertebral artery: a classification system for preoperative planning of C2 instrumentation based on 908 potential screw insertion sites. Spine J. 2025;25(1):59–68. 39255917 10.1016/j.spinee.2024.08.021

[9] Li J, Cao S, Guo D et al. Biomechanical properties of different anterior and posterior techniques for atlantoaxial fixation: a finite element analysis. J Orthop Surg Res. 2023; 18(1). 10.1186/s13018-023-03905-3 PMC10291817 37365580

[10] Lu M, Wang Z, Yuan B et al. The C2 isthmus screw provided sufficient Biomechanical stability in the setting of atlantoaxial dislocation-a finite element study. BMC Musculoskelet Disord. 2024; 25(1). 10.1186/s12891-024-07470-6 PMC11138020 38811940

[11] Lu Y, Hang G, Feng Y et al. Biomechanical comparison of anterior axis-atlanto-occipital transarticular fixation and anterior atlantoaxial transarticular fixation after odontoidectomy: A finite element analysis. Front Bioeng Biotechnol. 2023; 11. 10.3389/fbioe.2023.1067049 PMC10027935 36959905

[12] Brolin K, Halldin P. Development of a finite element model of the upper cervical spine and a parameter study of ligament characteristics. Spine. 2004;29(4):376–85. 15094533 10.1097/01.brs.0000090820.99182.2d

[13] Wang Xd F, Ms HY. Establishment and finite element analysis of a Three-dimensional dynamic model of upper cervical spine instability. Orthop Surg. 2019;11(3):500–9. 31243925 10.1111/os.12474 PMC6595113

[14] Ni M, Wong DW-C, Niu W, et al. Biomechanical comparison of modified calcanail system with plating fixation in intra-articular calcaneal fracture: A finite element analysis. Med Eng Phys. 2019;70:55–61. 31229383 10.1016/j.medengphy.2019.06.004

[15] Panjabi M, Dvorak J, Duranceau J, et al. Three-dimensional movements of the upper cervical spine. Spine. 1988;13(7):726–30. 3194778 10.1097/00007632-198807000-00003

[16] Panjabi MM, Crisco JJ, Vasavada A, et al. Mechanical properties of the human cervical spine as shown by three-dimensional load-displacement curves. Spine. 2001;26(24):2692–700. 11740357 10.1097/00007632-200112150-00012

[17] Cai X, Yu Y, Liu Z, et al. Three-dimensional finite element analysis of occipitocervical fixation using an anterior occiput-to-axis locking plate system: a pilot study. Spine. 2014;14(8):1399–409. 10.1016/j.spinee.2013.08.025 24231055

[18] Zhang B, Liu H, Cai X, et al. Biomechanical comparison of modified TARP technique versus modified goel technique for the treatment of Basilar invagination: A finite element analysis. Spine. 2016;41(8):E459–466. 26630424 10.1097/BRS.0000000000001297

[19] Xu S, Ruan S, Song X, et al. Evaluation of vertebral artery anomaly in Basilar invagination and prevention of vascular injury during surgical intervention: CTA features and analysis. Eur Spine J. 2018;27(6):1286–94. 29288330 10.1007/s00586-017-5445-4

[20] Dmitriev AE Jr, Helgeson RAL. Acute and long-term stability of atlantoaxial fixation methods: a Biomechanical comparison of pars, pedicle, and intralaminar fixation in an intact and odontoid fracture model. Spine. 2009;34(4):365–70. 19214095 10.1097/BRS.0b013e3181976aa9

[21] Sim HB, Lee JW, Park JT, et al. Biomechanical evaluations of various c1-c2 posterior fixation techniques. Spine. 2011;36(6):E401–407. 21372651 10.1097/BRS.0b013e31820611ba

[22] Chun DH, Yoon DH, Kim KN, et al. Biomechanical comparison of four different atlantoaxial posterior fixation constructs in adults: A finite element study. Spine. 2018;43:E891–7. 29462065 10.1097/BRS.0000000000002584

[23] Elliott RE, Tanweer O, Boah A, et al. Comparison of safety and stability of C-2 Pars and pedicle screws for atlantoaxial fusion: meta-analysis and review of the literature. J Neurosurg Spine. 2012;17(6):577–93. 23039110 10.3171/2012.9.SPINE111021

[24] Chang C-C, Huang W-C, Tu T-H, et al. Differences in fixation strength among constructs of atlantoaxial fixation. J NEUROSURGERY-SPINE. 2018;30(1):52–9. 10.3171/2018.6.SPINE171390 30485212

[25] Bydon M, Mathios D, Macki M, et al. Accuracy of C2 pedicle screw placement using the anatomic freehand technique. Clin Neurol Neurosurg. 2014;125:24–7. 25080046 10.1016/j.clineuro.2014.07.017

[26] Xu P, Lin J, Xiao H, et al. Feasibility of C2 pedicle screw fixation with the in-out-in technique for patients with Basilar invagination. Spine. 2024;49(11):798–804. 37339267 10.1097/BRS.0000000000004757

[27] Chen Z, Huang X, Zou X, et al. Feasibility and safety of the C1 zero angle screw: A novel In-Out-In technique for atlantoaxial dislocation. Orthop Surg. 2025;17(2):437–45. 39628069 10.1111/os.14309 PMC11787991

[28] Jian Q, Hou Z, Zhao X, et al. A screw algorithm for congenital C2-3 fusion with high-riding vertebral arteries: feasibilities and clinical outcomes of five different fixation techniques. Neurosurg Rev. 2024;47(1):520. 39222163 10.1007/s10143-024-02719-z

[29] Ouyang B, Zou X, Luo C, et al. Finite element analysis of horizontal Screw-Screw crosslink used in C1-C2 pedicle Screw-Rod fixation. Med Sci Monit. 2021;27:e932026. 34903706 10.12659/MSM.932026 PMC8684241

[30] Zeng B, Ding M, Li L, et al. Axis screw parallel to the sagittal plane versus traditional pedicle screw in the treatment of atlantoaxial fixation: a finite element study. BMC Musculoskelet Disord. 2024;25(1):931. 39563305 10.1186/s12891-024-08047-z PMC11577731

